# Editorial: Management of Primary Obstructive Megaureter

**DOI:** 10.3389/fped.2019.00365

**Published:** 2019-09-04

**Authors:** Alberto Parente, Ciro Esposito

**Affiliations:** ^1^Pediatric Surgery Department, Reina Sofía University Hospital, Córdoba, Spain; ^2^School of Medicine and Surgery, University of Naples Federico II, Naples, Italy

**Keywords:** megaureter, primary obstructive megaureter, ureteroscopy (URS), children, endourologic treatment

Primary obstructive megaureter (POM) represents one of the most challenging dilemmas in pediatric urology today. Antenatal and postnatal ultrasonography has significantly altered the detection of POM in children; not only being useful for the diagnosis, but also for the follow-up term. Thus, monitoring of these patients will continue for many years. Its assessment remains essential for the pediatric urologist.

Diuretic renography with radiotracers has been used successfully to diagnose obstruction in patients with hydronephrosis. However, in many cases we may obtain inconclusive results. MRI is not a well-established test in infants, as it requires general anesthesia. The combination of clinical and radiological findings may presumably be the most effective diagnostic method nowadays.

It is well-known that the majority of the POMs may be managed conservatively (spontaneous remission rates of up to 85%), but the indications for surgical intervention are less well-defined remaining controversial. Many authors believe that the large number of complications of ureteral reimplantation in infants with POM should be translated into a decrease in the indications for surgical treatment. Therefore, laparoscopic or endourological treatments such as endoscopic high-pressure balloon dilatation, endoureterotomy or temporary double-J stenting, could increase surgical attitudes. These treatments must show to be minimally invasive and have few complications. In addition, they must ensure good long-term results, similar to ureteral reimplantation.

The aim of this Research Topic is to attract articles from experts in the field that allow us to answer any of these questions.

Ortiz et al. reports the long-term results of the endoscopic treatment of the obstructive megaureter by high-pressure balloon dilatation. This is the largest series published of this technique, with 100 patients. One of its authors was the first to develop the technique exposed (1). In addition, it is the longest follow-up series published. Even thought the paper shows an evolution of the technique (it is done without fluoroscopic control in the last children), and it is necessary to assess the learning curve of its authors, it is an extremely homogeneous series with very few variations in the surgical technique and in postoperative management.

In Gregorio Marañon Hospital's paper, high-pressure balloon dilatation approach of POM had a long-term success rate of 87.3%. Mean follow-up was 6.4 ± 3.8 years. Secondary VUR was found in 17 cases (21.5%), being successfully treated by endoscopic subureteral injection in 13 (76.4%). Only one patient has Clavien III complication. In the authors' opinion, endoscopic balloon dilatation has proved to be safe, feasible, and really less-invasive procedure.

Kassite et al. described the experience of four French centers in the treatment of POM in infants by high-pressure balloon dilatation of POM. All centers used double JJ stenting after dilatation. Despite being a multicenter study, there is homogeneity in the surgical technique and also the material used is very similar in all centers, which allows us to correctly assess the results. Forty-two ureters were treated for POM in 33 children. The average age was 14.7 months. After one endoscopic treatment, ultrasound improvements were found in 86% of ureters. Three cases required a second dilatation. Four patients (9%) required ureteral reimplantation for ultrasound worsening. No patient required surgical treatment due to clinical worsening or infections. The average age of follow-up was 24 months. However, post-operative reports showed 11 cases of grade II (26%) and 10 cases of grade IIIb Clavien-Dindo (23%).

Dekirmendjian and Braga analyzed the possible risk factors that could predict which patients would need surgery and which ones would experiment a spontaneous resolution. This prospective study collected prenatal hydronephrosis database from 2008 to 2017. They chose patients with POM without another associated disease. Primary outcomes explained surgical intervention or hydronephrosis resolution. Ureteral dilatation resolution or <7 mm at last follow-up was defined as spontaneous healing. Hundred-one patients were analyzed. The conclusions of the data analysis were that high-grade hydronephrosis, urinary tract infection, and ureteric dilation ≥14 mm were significant risk factors for surgical intervention. Furthermore, ureteral dilation <11 mm was significantly associated with POM resolution.

Romero shows a new tool for systematic review. This paper aims to analyze endoscopic treatment with high-pressure balloon dilatation for POM using the IDEAL method. IDEAL model is used as a tool for new techniques or important surgical modifications. It is a method with 5 steps: Idea, Development, Exploration, Assessment, and Long-term Study; and provides an aid to assess the usefulness of these surgical innovations (2). It is based on the opinion that a new medical or surgical development cannot be compared with the techniques used for the same problem in recent decades. So, we need to compare the different stage of the development of the technique if it meets the objectives set in each stage. In opinion of the author, IDEAL framework is an excellent tool in pediatric urology, due to the considerably low prevalence of patients and the variability between them in age or weight.

The analysis of the results of the systematic review of patients treated with endoscopic treatment for POM with high-pressure balloon dilatation that the technique up to date is in stage 2a (evaluation of short-term outcomes) and stage 2b (development). The available evidence demonstrates that endoscopic high-pressure balloon dilatation is an effective treatment for patients with POM, with a long-term success rate of 87.7% and low morbidity.

## Author Contributions

All authors listed have made a substantial, direct and intellectual contribution to the work, and approved it for publication.

### Conflict of Interest Statement

The authors declare that the research was conducted in the absence of any commercial or financial relationships that could be construed as a potential conflict of interest.
